# Supplementary material for: MetaRibo-Seq measures translation in microbiomes
Source: Nat Commun. 2020 Jun 29;11:3268. doi: 10.1038/s41467-020-17081-z (PMC7324362; doi:10.1038/s41467-020-17081-z)
Supplement: Supplementary file 10 — Supplementary Data 7 [file 41467_2020_17081_MOESM10_ESM.zip › File2/Confidence_VeryHigh_Taxonomy/91229_out.krona.html]

Javascript must be enabled to view this page.

members
magnitude
magnitudeUnassigned
count
unassigned
taxon
rank

91229\_out

10

10
10239
superkingdom

340016
10

SRS011140\_contig\_number\_31274SRS014728\_contig\_number\_19582SRS020220\_contig\_number\_20585SRS022621\_contig\_number\_8820SRS045715\_contig\_number\_36071SRS054079\_contig\_number\_12728SRS076764\_contig\_number\_31636SRS098612\_contig\_number\_10009SRS146894\_contig\_number\_10153SRS149078\_contig\_number\_18911
species
